# One Scaffold, Many Targets: Fully Modified RNA Nanostructures for Multi-Gene Silencing Beyond the Liver

**DOI:** 10.64898/2026.10.08.757482

**Published:** 2026-10-09

**Authors:** Hassan H. Fakih, Ashley Summers, Qi Tang, Rosemary Gagnon, Brianna Bramato, Raymond Furgal, Katherine Y. Gross, Jana H. Fakih, Dimas Echeverria, Hanadi Sleiman, Anastasia Khvorova

## Abstract

Therapeutic siRNAs are a major clinical modality for hepatic gene silencing, but efficient extrahepatic delivery and multi-gene modulation remain significant barriers. Nucleic acid nanotechnology offers programmable assembly, but conventional structures suffer from enzymatic instability *in vivo* . Here we report fully chemically modified RNA nanostructures (FMRNs)—a vehicle-free platform entirely composed of stabilized therapeutic nucleic acids that integrate structural precision with metabolic durability. Following systemic administration, FMRNs exhibit enhanced pharmacokinetics and extrahepatic accumulation, with *in vivo* behavior influenced greatly by hydrodynamic size rather than molecular weight. Using triangular and square geometries, we demonstrate programmable control over siRNA stoichiometry to achieve sustained, simultaneous silencing of up to four genes from a single entity. Nanostructure assembly alone drives productive delivery, especially to heart and muscle, matching lipophilic conjugation. Furthermore, cerebrospinal fluid administration reveals that larger FMRNs improve central nervous system distribution and retention revealing physicochemical parameters that impact brain exposure. FMRNs establish a modular and effective platform combining improved pharmacokinetics, extrahepatic delivery, and multi-gene silencing for complex diseases.

## Full Text Availability

The license terms selected by the author(s) for this preprint version do not permit archiving in PMC. The full text is available from the preprint server.

